# Estimating the future health and aged care expenditure in Australia with changes in morbidity

**DOI:** 10.1371/journal.pone.0201697

**Published:** 2018-08-09

**Authors:** Anthony Harris, Anurag Sharma

**Affiliations:** 1 Centre for Health Economics, Monash Business School, Monash University, Melbourne, VIC, Australia; 2 School of Public Health University of New South Wales, Sydney, NSW, Australia; James Cook Unviersity, AUSTRALIA

## Abstract

**Aims:**

We estimate the pure effect of ageing on total health and aged care expenditure in Australia in the next 20 years.

**Methods:**

We use a simple demographic projection model for the number of people in older age groups along with a needs based estimate of changes in the public and private cost of care per person in each group adjusted for expected changes in morbidity.

**Results:**

A pure ageing model of expenditure growth predicts an increase in health expenditure per elderly person from $7439 in 2015 to $9594 in 2035 and an increase in total expenditure from $166 billion to $320 billion (an average annual growth of 3.33%). If people live longer without additional morbidity, then total health expenditure only grows at an average annual rate of 0.48%. If only some of those additional years are in good health, then the average year on year growth is 1.87%.

**Conclusion:**

Ageing will have a direct effect on the growth of health spending but is likely to be dwarfed by other demand and supply factors. A focus on greater efficiency in health production and finance is likely to be more effective in delivering high quality care than trying to restrain the demand for health and aged care among the elderly.

## Introduction

There is a popular perception in many developed countries that ageing of the population will increase the cost of health services to the point where it challenges the willingness of the public to continue to subsidise high quality health care for everyone. This paper estimates the effect of changes in the age distribution of the population on the cost of health and aged care in Australia in the medium term (next 20 years). In particular, we focus on changes in the cost per person directly due to ageing, the share of rising total costs caused by increased life expectancy, and factors that might restrain the age related increase in health and aged care costs.

**Fig 1:**
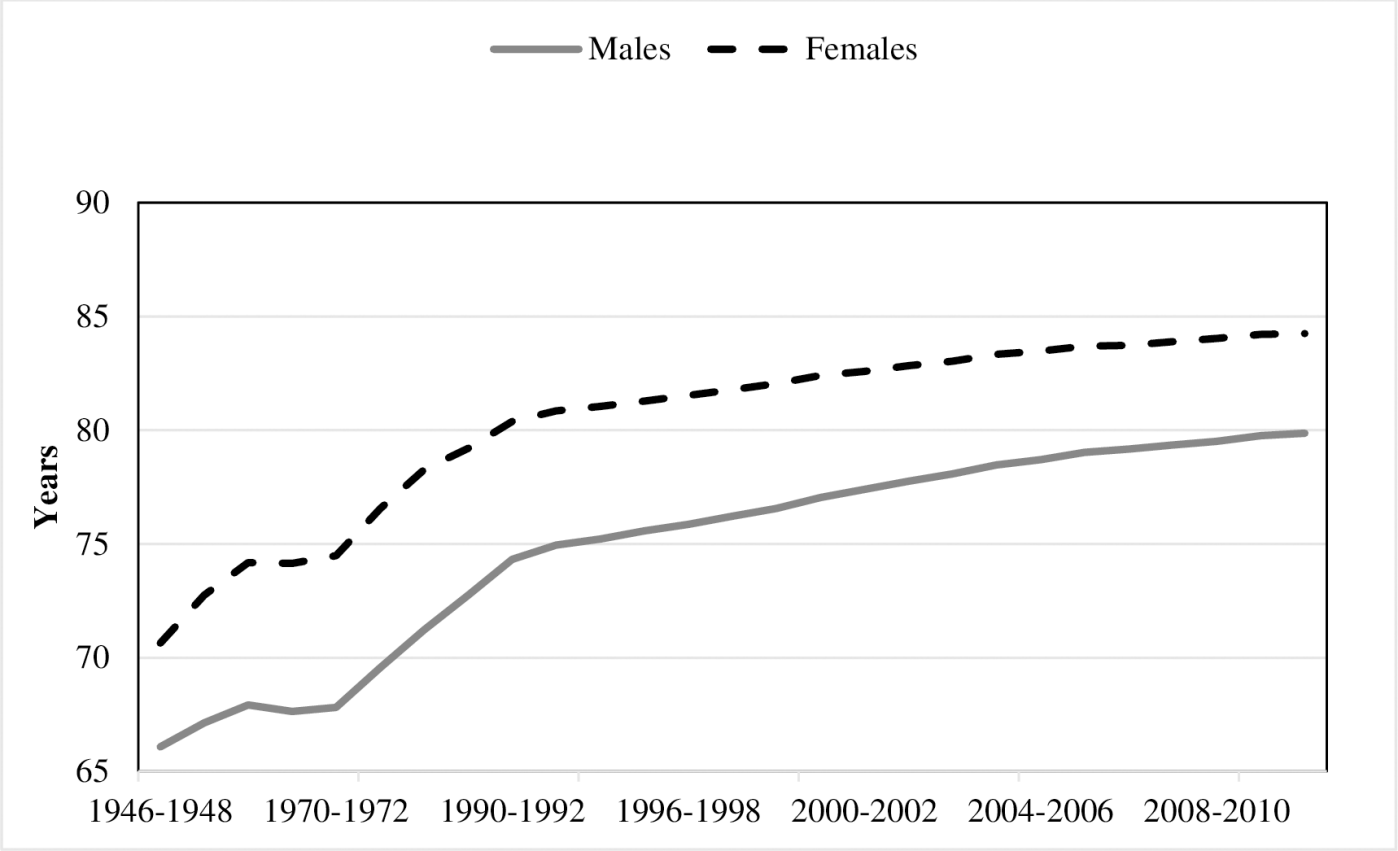
Life expectancy at birth. Source: Australian Historical Population Statistics 2014 ABS cat. no. 3105.0.65.001).

Economic development, along with advances in medical technology, has resulted in large increases in the quality and length of life across the industrialised world in the last 30 years. In Australia we have seen remarkable increases in life expectancy in the last 50 years (Fig 1) along with a dramatic fall in fertility since the end of the baby boom in the late 1960s. Together these developments have meant that the proportion of the population that is elderly will increase in the coming decades. Since the elderly currently have a higher health cost per capita it seems reasonable to assume that their share of the health expenditure will rise in coming decades.

By 2063 the population of Australia is projected to be 42 million. On this projection the proportion of people aged 65 and over will rise from 15% in 2014 to 23% in 2063. The working age population (15–64) will decrease from 66% to 61% of the population, and the age dependency ratio will be 28% [1].

One might expect *a priori* that an ageing population would be associated with an increase in health care expenditure per person. If the share of older people in the population is growing faster than that of any other age group, both as a result of longer lives and a lower birth rate, an automatic increase in the average health expenditure seems inevitable However, this intuition finds little support in the empirical literature. Assessing the effect of population ageing on health and health care has proved to be far from straightforward [2] and problematic to extrapolate into the future, The long term econometric evidence from many countries generally suggests that pure ageing (longevity) is not a major contributor to the historical growth in per capita health expenditure but that service volume and unit cost are the main contributors [3]. In Switzerland for example Breyer et al [2] find that population ageing accounts for only a 0.5 percent annual growth rate of health-care expenditure. Rossen and Faroque [4] conclude that much of the growth of health care spending in Canada has been driven by progress in medical technology and increasing per capita income. The process of ageing has been just too slow to explain much of the historical growth in annual per capita expenditure on health in countries in Europe, Canada or Australia [5]. The pure effect of ageing on health spending has been so weak that as source of policy concern it has been somewhat contentiously described as a “red herring” [6]. Of course even if pure ageing has not been the main cause of health expenditure growth in the past, it may be that more substantial changes in demographic structure combined with increased longevity might put pressure on future total health care costs [7].

In this paper we ignore other causes of health care expenditure growth and any market or non-market responses that might mitigate growth, focussing instead on the direct contribution of an ageing population to health and aged care expenditure in the coming decades. In effect we ask the restricted question of to what extent is ageing *per se* likely to be a major determinant of the future growth of health and aged care expenditure per person. In the discussion we return to the alternative explanations of expenditure growth and future behavioural, market and policy responses to that growth.

## Materials and methods

### Health expenditure

Total health expenditure growth arises from an increase in prices (potentially associated with quality or cost), an increase in service volume per capita of treatment, an increase in population, or some combination of these three elements. A redistribution of private and public health expenditure towards the elderly could happen only if a) there is a greater number of older people with a higher than average cost of care or b) the cost per case rises faster in the elderly.

Any prediction of the effect of ageing on health care expenditure needs to distinguish between increased life expectancy and demographic change i.e. between the effect of the average person living longer and potentially using more resources (because of illness) and change in the number of people at each age. As Cutler et al [8] usefully categorise it, health and care expenditure at any time is the sum over all ages of the product of (1) the number of people alive in each group (2) the average health status at each age and (3) the per capita spending conditional on health status, which also varies according to age.

We use three alternative empirical models to make predictions for the next 20 years of demographic induced:

- health expenditure per elderly person;
- total healthcare expenditure on the elderly (those aged 65 years and over) and;
- total health expenditure.

The approach is similar to that in Caley and Sidhu [9].

### Model 1

This is the most basic model which derives predicted healthcare expenditure as a product of age and gender specific per capita health care costs and population projection corresponding to that age and gender. We initially use per capita health cost by age and gender at 2015 prices and multiply it with 2015 population by age and gender to get total health expenditure for 2015. We then change the age-gender distribution to that which is forecast to prevail in next 20 years till 2035 and multiply the average expenditure of each age group by the new age distribution for each year. Age groups are 0–4, then 10 year age bands to 84, and 85 and over. All other factors are assumed fixed, (including Gross Domestic Product, quality and volume of care and average health care costs for a given age).

### Model 2

One drawback of Model 1 is that it assumes the annual health care cost of say 80 year old today will be the same in next twenty years. However, this might not be the case. There is evidence to suggest that health care costs for elderly people are more strongly related to their proximity to death rather than their calendar age. [10] This proximity will change across cohorts due to changes in life expectancy. An 80 year old today will be relatively closer to death compared to an 80 year old 20 years from now due to increased life expectancy. Ignoring this will overestimate future annual health care expenditure predictions. Model 2 makes a simple adjustment to the annual health care expenditure projection for changes in life expectancy. We attribute the cost of care of an earlier age group to the actual age. We use annual increase in life expectancy of 0.25 and 0.19 years for males and females respectively as in the Australian Bureau of Statistics (ABS) Series B projections. This adjustment will result in a slower rate of annual growth in health expenditure compared to that derived from Model 1.

### Model 3

The main assumption in model 2 is that morbidity is delayed by the same amount of time as the increase in life expectancy. This is a reasonable assumption if all health costs in Fig 1 are simply shifted to the left but not if the additional years of life result in greater exposure to additional illnesses. In model 3 we adjust the change in life expectancy for changes in morbidity (expansion or compression) using a measure of disability free life expectancy. Dividing the increase in disability by the increase in life expectancy provides the proportion of gain in healthy life expectancy and a proxy for the years free of morbidity and higher health care costs. Based on the data ABS survey data the Australian Institute of Health and Welfare (AIHW) estimated that in 2009 44% of the years beyond age 65 were disability free.[11] Based on this we assume that there will be an expansion of morbidity with 44% of the extra years spent free of disability. This figure is then used to adjust the age bands in Model 2 by multiplying increase in the life expectancy by 0.44. The value of estimates derived from model 3 lie between those derived in model 1 and model 2.

The first model uses age specific public and private health care cost obtained from data reported by AIHW and applies it to projected population from 2015 to 2035 from the ABS series B projection which uses the cohort-component method to predict future population. This method assumes a total fertility rate of 1.8 births per woman by 2025–26; life expectancy increasing to reach 85.2 years for men and 88.3 years for women by 2061; and net overseas migration of 240,000. The annual population growth rate is projected to be 1.7% from 2010, although we note that the latest growth rates for 2014–2015 are lower than this at 1.4%.

The second model adjusts the age specific health care cost for life expectancy and the third model projects future health care costs by including adjustments for both life expectancy and changes in morbidity.

We use estimates of expenditure by disease category, age group and sex for: admitted patient hospital services, out-of-hospital medical services, prescription pharmaceuticals, optometric and dental services, community mental health services and public health cancer screening [12] to derive proportions of disease expenditure by age and gender. We have calculated health expenditure by age by allocating total health expenditure [13] to age groups based on weights by age from 2008–09 disease allocations (AIHW 2010). These estimates are then converted to 2015 prices by using the ABS Health Price Index. This ensures that we have captured all health costs public and private including out of pocket costs, non-subsidised clinical treatments and therapies and over the counter drugs. A limitation of this approach is that the attribution by age group is based on an earlier study of disease costs that attributes costs on the basis of disease prevalence. It is not clear that this accurately reflects the pattern of actual expenditure.

In a separate analysis we predict total expenditure on aged care services in the next twenty years. Aged care historically has been provided through three major programs in Australia: i) Residential aged care in an aged care facility which in 2012–13 costs government around $54,362 per resident each year; ii) Extended aged care at Home (EACH) program which cost government $44,410 and $48,494 per non-dementia and dementia patient respectively and iii) Home and Community care program (HACC) which cost government $13,517 per person.[14] In addition many individuals make contributions to their care (reported by the Henry review (2010) as 26% to 53% of the total for residential low and high care places). Those who live in their own home pay for their own living costs (including implicit rent) some of which comes from state pension income. In the absence of accurate information on these components of total cost, we use the government recurrent expenditure figures as estimates of the average cost of care by need category along with population projections for those aged 65 years and over to predict total expenditure on care in the next twenty years. It is not clear living costs increase or fall with age nor how that pattern will change in the coming decades. In this study we ignore personal living costs associated with ageing in the community and focus on the cost to government of caring for the elderly in residential aged care facilities. We use population projections as before, attributing the 2012–13 cost of care per person in residential care. In parallel to model 3 above we adjust for the increase in longevity and reduced morbidity by assuming that annual entry to care is delayed by 44% of the increase in longevity.

As a robustness check on all predictions we make alternative assumptions on longevity and fertility changes in the next 20 years. We used the Productivity Commission’s approach [15] to generate population projections under three scenarios of low and high fertility rates with higher longevity and found no significant change in our expenditure projections.

## Results

**Fig 2:**
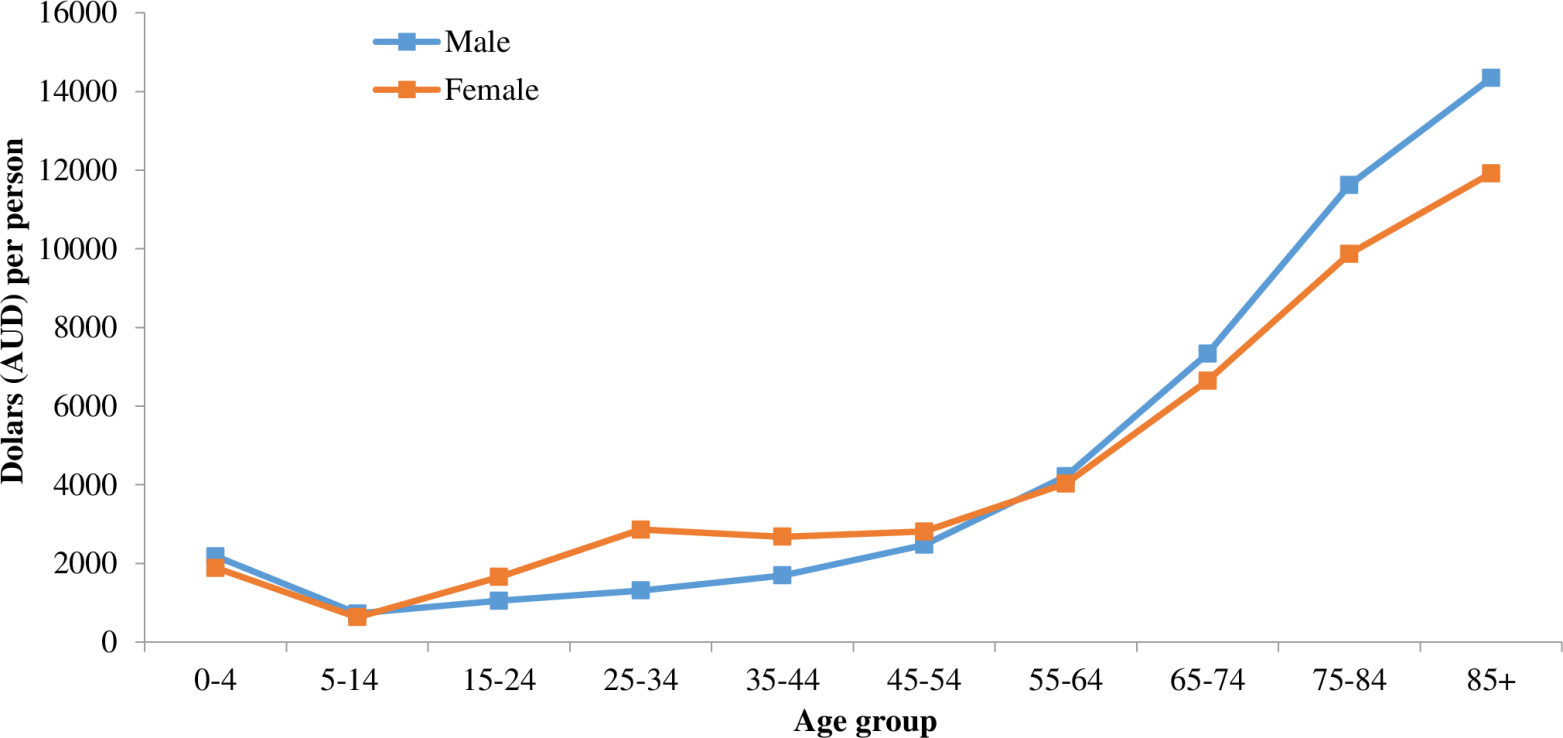
Total health expenditure 2008–09 per person, allocated by age. Source: Australian Institute of Health and Welfare. Australia's health 2014. Australia's health series no. 14. Cat. no. AUS 178. Canberra: 2014.

The average annual health care costs for a 1 year old male and female are $4780 and $4249; $2814 and $6258 for 25 year old males and females; and $25380 and $22757 for 80 year old males and females. The estimated pattern of health care expenditure by age and gender is shown in Fig 2.

The ABS projections of the population for next 20 years show the average annual growth rate in elderly population will be 2.76% compared to 1.2% in the cohort aged below 65 years. The proportion of elderly population is projected to increase from 14% in 2016 to 18% in 2035 for males and from 16% to 20% for females, although the average annual rate of increase will slow down post 2021 from 1.6% to 0.82%.

### Illustrative predictions

**Fig 3:**
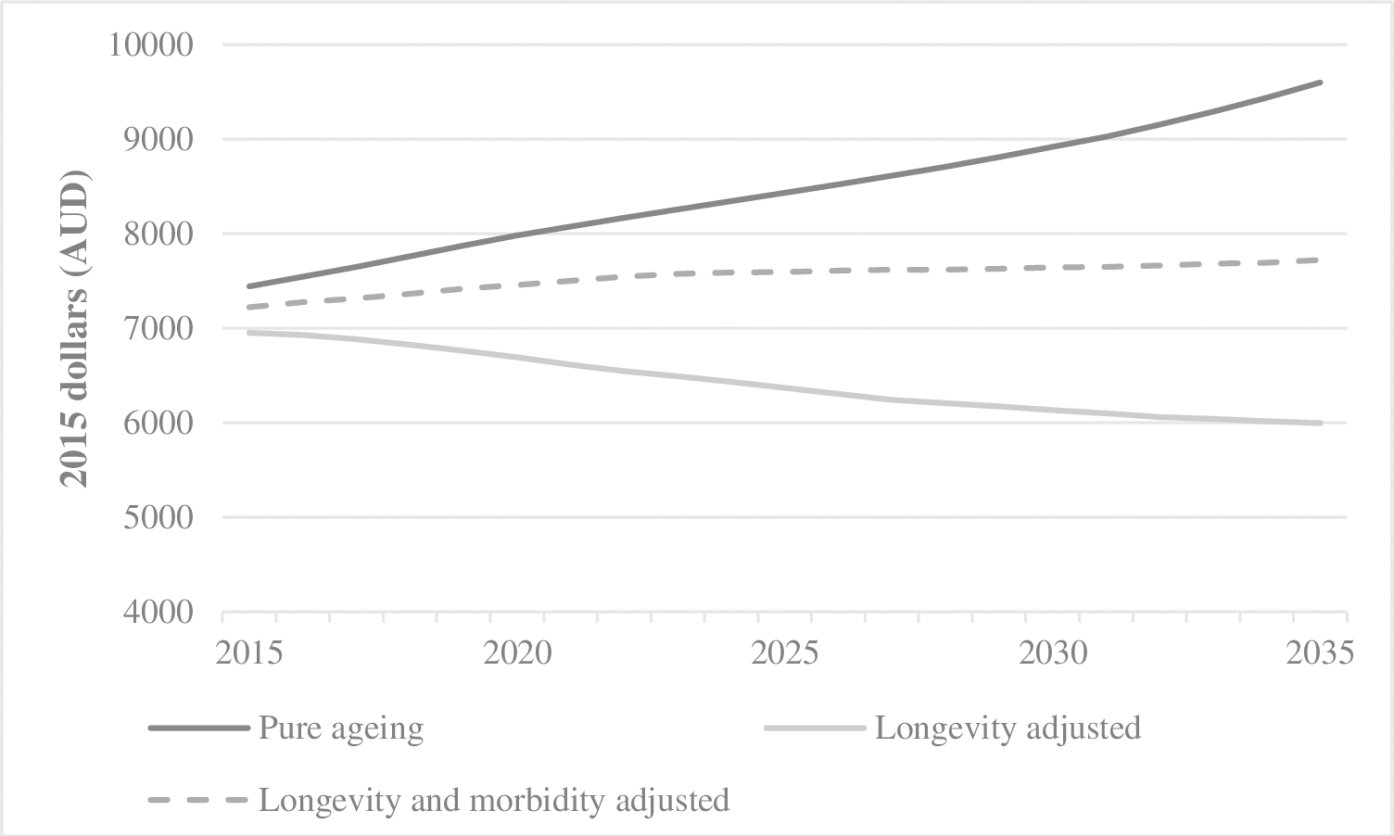
Age related projected health expenditure per person for those over 65 years of age.

**Fig 4:**
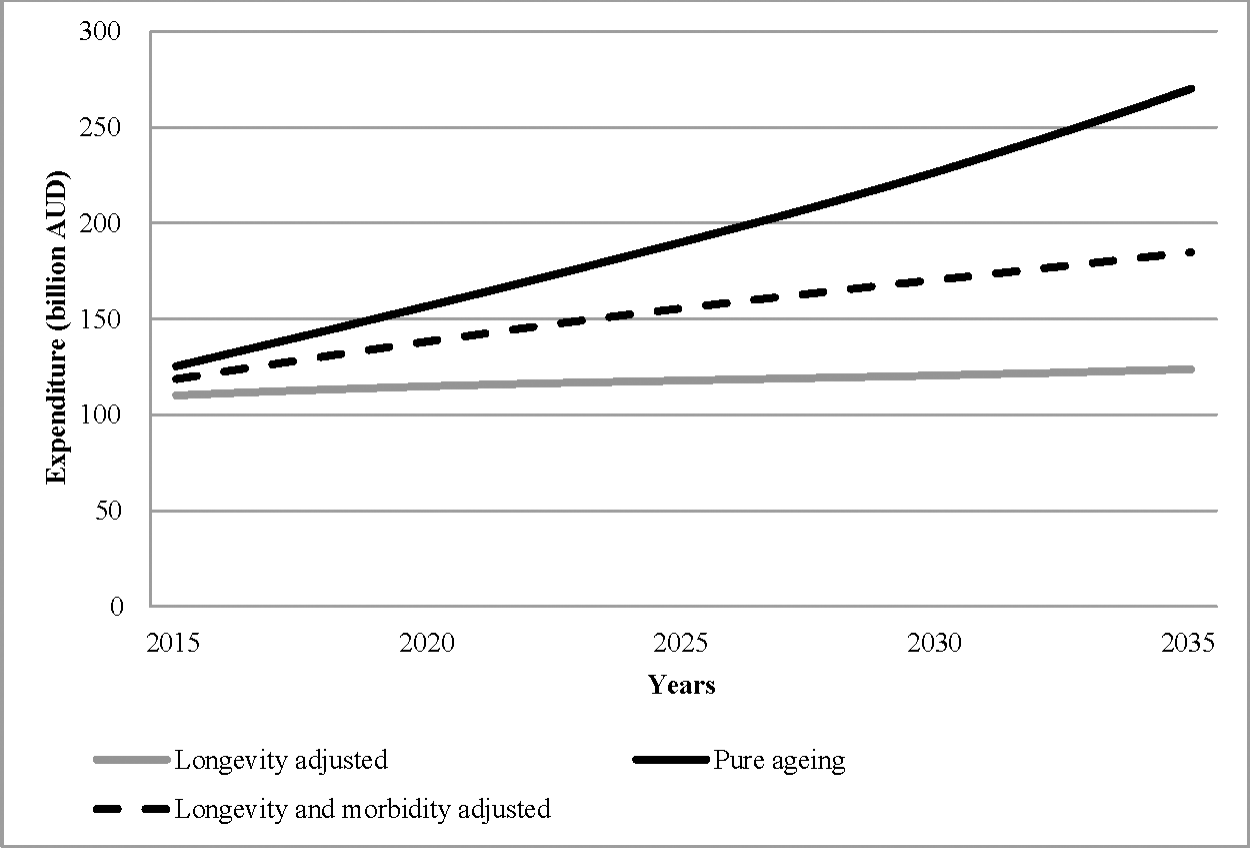
Age related projected health expenditure for those over 65 years of age.

The yearly predictions for demographic induced expenditure per elderly person and total health expenditure on the elderly, based on the three different models, are shown in Fig 3 and Fig 4.

The pure ageing model of expenditure growth (model 1) predicts an increase in health expenditure per elderly person from $7439 in 2015 to $9594 in 2035, an increase in total health expenditure on the elderly from $125 billion to $270 billion (average annual growth of 3.9%), and an ageing related increase in total health expenditure from $166 billion in 2015 to $320 billion in 2035 (average annual increase of 3.3%). Allowing for an improvement in life expectancy (model 2), with no additional morbidity in those extra years, predicts a fall in heath expenditure per elderly person from $6951 to $5994, an increase in total health expenditure on the elderly from $110 billion to $124 billion (average annual growth of 0.58%), and an increase in total health expenditure from $152 billion in 2015 to $168 billion in 2035 (average annual growth of 0.48%). Adjusting for both life expectancy and morbidity (model 3) predicts an increase in health expenditure per elderly person from $7220 to $7719, an increase in total health expenditure on the elderly from $110 billion to $124 billion (average annual growth of 0.58%), and an increase in total health expenditure from $162 billion in 2015 to $232 billion in 2035 (average year on year growth of 1.87%). The demographic driven change in health expenditure is thus a consequence of a change in the distribution of the population towards the elderly and a change in their expenditure per person at each age. Different assumptions on these two factors mean not only substantial differences in total expenditure but also in the share of health expenditure spent on the elderly. For example, model 1 predicts an increase of 10 percentage points in the share of total health expenditure for those aged 65 and over (74% in 2015 to 84%); model 3 predicts an increase in the share of 6 percentage points; while model 2 predicts an increase of only 2 percentage points.

Demographic change in the next 20 years, although by no means the main driver of health expenditure growth, will be associated with a significant change in the structure of the spending. However even this projected growth may be further mitigated by improvements in longevity and reductions in age related morbidity over time.

The results for aged care services show a consistent average year on year growth of around 3.55% doubling total expenditure from $11 billion in 2015 to around $23 billion in 2035. While this represents a high growth rate it is not a significant component of overall health and aged care expenditure. Indeed, as a proportion of total health expenditure on the elderly, expenditure on residential aged care is predicted to decrease slightly from 9.6% in 2015 to 8.6% in 2026 before moving up to 8.8% in 2035.

## Discussion

Separating out these influences is important for the public policy response. If we believe that future expenditure growth is largely caused by changing demographics, we might consider policies targeted at the elderly, for example, increasing the efficiency of the delivery of care for chronic disease. If on the other hand the rise in expenditure is due more to exogenous technical change then we might focus on assessing the effectiveness and cost effectiveness of new technology in improving the quality of care. If we believe it is largely the result of illnesses or behaviours that are preventable we might focus on targeted interventions that reduce the risk of chronic illness. Of course these are not mutually exclusive, but we should be conscious of the risk of introducing policy measures that focus on one of these suspected causes that may have adverse effects on one of the others. For example, constraining demand for care through direct patient payments or insurance requirements may have perverse effects on health outcomes while not significantly slowing expenditure growth and having consequences for health equity.

Even in the absence of public policy to change incentives for workforce participation among older people, we might expect increased longevity to change behaviour with respect to consumption and labour supply with people being more likely to enter the labour market, to increase savings, be more productive and remaining in the labour market for longer. In terms of the effect of longer working lives on health it is unclear what the impacts of these changes will be. A longer attachment to the labour force may improve mental and physical health for some, but for others in less amenable jobs it may reduce future physical health.

The actual size of the change in composition of health expenditure towards the elderly will depend not just on the change in population numbers by age, but how the elderly, care providers and government respond in the future. An expectation of rising wealth among elderly will lead providers to offer additional or higher quality services. To the extent that that people can be induced to use these services at similar or higher cost we might expect to see greater expenditure per capita. In addition, we might expect to see political lobbying from the elderly or providers to subsidise these new technologies or to provide additional income to pay for them. These indirect effects of an expansion of the elderly in the population may well be more powerful in increasing health and aged care expenditure than the demographic effects that we have discussed so far.

If the projected compositional change in expenditure in the next 20 years comes to pass there is a political question of the willingness to continue a tax based social insurance system that redistributes current consumption between younger and older cohorts in the population. In 2012, 71% of health expenditure was directed to people aged 65, and over, with these persons representing 14% of the population. In 2035, nearly 84% of health expenditure will be directed to people aged 65 and over while they will represent 19% of total population. This has led to discussion of proposals to change the way in which health and aged care are financed with a shift towards self-financing through subsidised saving and later retirement. However there are clearly potential problems meeting the equity objectives of a universal health care system with greater personal payments from the elderly on the one hand and subsidising savings among those with high income and wealth[16]. It is possible that behaviour in the market will achieve some of this irrespective of policy change as people save more and work longer to pay for their expected living costs in longer lives. In a recent paper Kudrna *et al* [17] use a lifecycle behavioural approach within a very long term macroeconomic general equilibrium economic model to allow for responses to demographic change. They assume that people will supply more labour (increased working hours, stay longer in the labour force) to pay for their longer lives, and then save more for their retirement. In world of perfect markets (and with no exogenous technical change or investment driven by ageing) the result would be that wages and Gross Domestic Product would fall while assets held by the elderly would rise. In other words, national income and a change in longevity are not independent and in the absence of major policy change we might expect to see adjustments in behaviour that affect future expenditure and the distribution of assets across generations. There is an opportunity cost in health care for the elderly insofar as those resources could be used by younger people. Consumption expenditure by the elderly at a point in time represents an intergenerational transfer of resources that could become an economic or political problem in the future. While it is true that the elderly consumes more health and personal services per person and contribute less to production of goods and services than those of working age, they also consume less in general and retain accumulated assets (particularly housing) that in the longer run will be transferred to the next generation.

A major confounder of any attempt to predict future health care costs by age group from past behaviour is the expected general rise in the cost of care for many illnesses independent of age. A significant part of that increase has been from an increased use of technologies and treatments, often involving new more expensive tests, procedures and drugs. Although this will likely mean that later cohorts of the elderly will have higher costs, this is not the consequence of ageing *per se*. Not all health technologies are cost enhancing, but it is possible that the rising number of elderly with a perceived demand for care will induce new possibly more expensive treatments. It is possible that this technological change targeted for example at chronic diseases of the elderly will improve quality of life but result in higher average costs at older ages.

All of this suggests that appropriate policy settings on health and aged care expenditure cannot be derived from simple demographic models. To the extent that the growth in per person expenditure is largely driven by supply side factors, and only indirectly by demand, then policies that focus on saving and the demand for care will be counterproductive. If the main cause of health growth continues to be technology and producer interests then increasing savings and wealth or encouraging savings for health expenditure will result in greater expenditure, not necessarily better health, and no less future income transfers.

It is true that demographic change in the coming decades will be faster than we have experienced so far and ageing is likely to have a stronger effect, but there is no immediate crisis in health care that requires restraints on the demand for health and aged care among the elderly through major finance reform. A stronger focus on efficiency in health production and finance that focusses on supply side drivers of the growth in spending remains an effective strategy to deliver high quality care at an acceptable cost.

## Conclusions

We have made a number of spending projections for the next 20 years but whether any of them become a reality will depend on policy responses, attitudes to spending overall on health and aged care and in part on who pays for health and aged care. Research suggests that there is a considerable amount of savings to be made in reducing unnecessary and inappropriate health care [7] and it seems likely that there are opportunities in the health and aged care sectors to offset to a considerable degree any additional financial cost of ageing.
